# First detection and a new avian host of the tick *Ixodes ventalloi* Gil Collado, 1936, in Slovakia

**DOI:** 10.1007/s00436-024-08286-y

**Published:** 2024-07-12

**Authors:** Katarína Loziaková Peňazziová, Lidia Chitimia-Dobler, Tomáš Csank, Branislav Peťko, Anna Ondrejková, Miloš Halán, Petra Schusterová, Soňa Pivka, Ľuboš Korytár

**Affiliations:** 1 Department of Microbiology and Immunology, University of Veterinary Medicine and Pharmacy in Košice, Komenského 73, 041 81 Košice, Slovakia; 2 Bundeswehr Institute of Microbiology, Neuherbergstr. 11, 80937 Munich, Germany; 3 Fraunhofer Institute of Immunology, Infection and Pandemic Research, Penzberg, Germany; 4 Department of Epizootiology, Parasitology and Protection of One Health, University of Veterinary Medicine and Pharmacy in Košice, Komenského 73, 041 81 Košice, Slovakia

**Keywords:** *Ixodes ventalloi*, *Prunella modularis*, Slovakia, Avian migration

## Abstract

This study describes the first detection of *Ixodes ventalloi* in Slovakia. Two engorged females of *I. ventalloi* were collected from Dunnocks (*Prunella modularis*) captured in eastern Slovakia. The identification of females was based on morphological and molecular 16S rRNA gene features. Phylogenetic analysis revealed a classification of the females into distinct genogroups. Moreover, comparative morphological analysis highlighted variations between the two females, particularly in the curvature of the auriculae, the shape of coxa I, and the internal spur. These findings suggest the potential for varied phenotypes of *I. ventalloi* correlated with their genogroups. Nonetheless, *I. ventalloi* population establishment within Slovakia necessitates further investigation through flagging or drag sampling.

## Introduction

The genus *Ixodes* Latreille, 1795, belonging to the family Ixodidae Murray, 1877, encompasses a group of hard-bodied ticks found across all zoogeographic regions (Guglielmone et al. 2014). Certain members of this genus, particularly those within the *Ixodes ricinus* complex, are known for their significance in both medical and veterinary contexts (Estrada-Peña et al. 2017; Santos and Santos-Silva 2018). A lesser-known species is *I. ventalloi* Gil Collado 1936, characterized by its relatively small size and ventrally curved auriculae (Gil Collado 1936). This species is frequently mistaken for *I. festai* due to their similar morphological features. Consequently, the misidentification of *I. ventalloi* has led to ambiguities regarding its ecology, geographic distribution, and role as a vector of tick-borne pathogens (Estrada-Peña et al. 2018; Guglielmone et al. 2014; Santos and Santos-Silva 2018).

*Ixodes ventalloi* is a Palaearctic species. It is known in Cyprus, France, Tunisia, Morocco, Spain, Portugal, Italy, and Great Britain (Estrada-Peña et al. 1984; Chastel et al. 1984; Ioannou et al. 2009; Jameson and Medlock 2011; Mori et al. 2015; Otranto et al. 2014; Pennisi et al. 2015; Santos-Silva et al. 2011). *Ixodes ventalloi* is considered a three-host, endophilic, and monotropic tick (Estrada-Peña et al. 2018; Santos and Santos-Silva 2018). Historically, it has been associated with host specificity, primarily targeting lagomorphs on which all developmental stages parasitize, hence its designation as the “rabbit tick” (Estrada-Peña et al. 2017; Gilot and Perez 1978; González et al. 2016; Jameson and Medlock 2011). However, its host range is more extensive and includes carnivores, rodents, birds, and even humans (Gil Collado 1936; Gilot and Perez 1978; Ioannou et al. 2009; Martínez-Carrasco et al. 2007; Millán et al. 2007; Mori et al. 2015; Otranto et al. 2014; Pennisi et al. 2015; Santos et al. 2004; Santos and Santos-Silva 2018; Santos-Silva et al. 2006).

Although the vectorial capacity of *I. ventalloi* has not been confirmed under laboratory conditions, it is speculated to be a mega-vector, similar to *I. ricinus* (Santos and Santos-Silva 2018). Despite being relatively understudied, *I. ventalloi* is associated with at least 13 pathogenic agents, including viruses such as the Eyach and Erve viruses (Estrada-Peña et al. 2017; Chastel et al. 1984), bacteria like *Anaplasma phagocytophilum* (Santos et al. 2004, 2018), *Rickettsia helvetica* (Márquez and Millán 2009; Santos-Silva et al. 2006), and *Coxiella burnetii* (Ioannou et al. 2009; Psaroulaki et al. 2014), as well as protozoans including *Leishmania infantum* (Pennisi et al. 2015) and *Theileria annulata* (Antunes et al. 2016). These pathogens have been detected not only in questing ticks collected from vegetation but also in those feeding on wild animals, domestic cats, and humans (Antunes et al. 2016; Chastel et al. 1984; Ioannou et al. 2009; Latrofa et al. 2017; Márquez and Millán 2009; Pennisi et al. 2015; Santos et al. 2004, 2018; Santos-Silva et al. 2006).

In this manuscript, we describe the first detection of *I. ventalloi* in the Slovak Republic. *Ixodes ventalloi* females were collected from two Dunnocks (*Prunella modularis*); to our knowledge, this is also the first record of *I. ventalloi* parasitization on this bird species.

## Material and methods

### Capturing of birds and tick collecting

Bird capturing was carried out on the Bird Ringing Station Drienovec, which is located in the Drienovská wetland in Southeast Slovakia. The geographical coordinates of the site are 48°37′ N, 20°55′ E.

Birds were captured and handled by a licensed ornithologist and a member of the ringing station team (Ľ.K.) under the Permission No. 3320/2019–6.3 from Act. No. 543/2002 of the code on nature and landscape protection, granted by the Ministry of Environment of the Slovak Republic. Birds were trapped during spring ringing campaign in 2021.

Ticks were removed from birds using a tick removal spoon (Dr. Kapiller®, Budapest, Hungary).

### Tick identification

Ticks were identified based on morphological identification keys (Estrada-Peña et al. 2018; Pérez-Eid 2007), under a Keyence VHX‑900F microscope (Itasca, IL, USA).

### DNA extraction, amplification, and sequence analysis

DNA was extracted from individual ticks using the QIAamp mini DNA extraction kit (Qiagen, Hilden, Germany) according to the manufacturer’s instructions. The 16S rRNA gene was amplified according to Halos et al. (2004) and subsequently sequenced.

The obtained reads of the partial 16S rRNA genes were de novo assembled and mapped to reference sequences by Geneious 9.1.8 software (Biomatters, Auckland, New Zealand) downloaded from GenBank. In the case of sample 438_16S (Female A) and sample 439_16S (Female B), 291 bp and 299 bp long consensus sequences were obtained, respectively. These sequences were used in Clustal Omega 1.2.2 (Sievers et al. 2011) alignment with partial 16S rRNA gene *Ixodes ventalloi* sequences downloaded from GenBank. The nucleotide sequences of samples 438_16S and 439_16S were deposited in GenBank with accession numbers: PP301986 (438_16S) and PP301987 (439_16S).

The best-fit substitution model and the phylogenetic tree were built using the MEGA 11 (Tamura et al. 2021). The evolutionary history was inferred by using the Maximum Likelihood method and General Time Reversible model (Nei et al. 2000). The percentage of trees in which the associated taxa clustered together is shown next to the branches. Initial tree(s) for the heuristic search were obtained automatically by applying Neighbor-Join and BioNJ algorithms to a matrix of pairwise distances estimated using the Maximum Composite Likelihood (MCL) approach and then selecting the topology with superior log likelihood value. A discrete Gamma distribution was used to model evolutionary rate differences among sites (5 categories (+ G, parameter = 0.5243)). The tree is drawn to scale, with branch lengths measured in the number of substitutions per site. This analysis involved 48 nucleotide sequences of *I. ventalloi* collected in Italy, Portugal, and Slovakia and other related *Ixodes* species available in GenBank. There was a total of 205 positions in the final dataset.

### Quantitative characteristics of the captured bird population

Using the ecological index of dominance (IED%), we characterized the population of captured birds (Margolis et al. 1982; Zając et al. 2022):

$$\mathrm{IED}=\frac{\mathrm{NSB}}{\mathrm{NB}}\times 100\mathrm{\%}$$

IED is the ecological index of bird species dominance, NSB is the number of birds of a particular species, and NB is the total number of birds.

According to previous studies (Bush et al. 1997; Margolis et al. 1982; Zając et al. 2022), the prevalence of tick infestation in a bird species (PTI%) and the mean intensity of tick infestation in a certain species (MIT) were calculated:

$$\mathrm{PTI}=\frac{\mathrm{NSBT}}{\mathrm{NSB}}\times 100\mathrm{\%}$$

PTI is the prevalence of tick infestation per bird species, NSBT is the number of birds of a particular species infested with ticks, and NSB is the number of birds of a particular species

$$\mathrm{MIT}=\frac{\mathrm{NTSB}}{\mathrm{NSBT}}$$

MIT is the mean intensity of tick infestation per bird, NTSB is the number of all tick species collected from a particular bird species, and NSBT is the number of birds of a particular species infested with ticks.

## Results

### Ornithological and parasitological findings

During two bird-trapping expeditions in March 2021, a total of 41 birds, belonging to eight species, were captured. Based on IED data, European Robins (*Erithacus rubecula*) represented the predominant species, accounting for 60% (*n* = 25) of the total capture, followed by the Common Blackbird (*Turdus merula*) at 14.6% (*n* = 6) (Table 1).

**Table 1:** List of captured bird species, tick infestation, and tick species

Bird species	NSB	IED (%)	MS	NSBT	NTSB	Tick species			PTI (%)	MIT
						***I. ricinus***		***I. ventalloi***		
						**L**	**N**	**A**		
*Cyanistes caeruleus*/Eurasian Blue Tit	2	4.9	S	**-**	**-**	**-**	**-**	**-**	**-**	**-**
*Emberiza cia*/Rock Bunting	1	2.4	S	**-**	**-**	**-**	**-**	**-**	**-**	**-**
*Emberiza schoeniclus*/Reed Bunting	2	4.9	S	**-**	**-**	**-**	**-**	**-**	**-**	**-**
*Erithacus rubecula*/European Robin	25	61	S	**-**	**-**	**-**	**-**	**-**	**-**	**-**
*Parus major*/Great Tit	2	4.9	S	1	4	-	4	-	50	4
*Prunella modularis*/Dunnock	2	4.9	S	2	5	-	3	2	100	2.5
*Turdus philomelos*/Song Thrush	1	2.4	S	**-**	**-**	**-**	**-**	**-**	**-**	**-**
*Turdus merula*/Common Blackbird	6	14.6	S	5	10	1	9	-	83.3	2

Legend: *NSB*, number of birds of a particular species; *IED*, index of ecological dominance of bird species; *MS*, migratory status; *S*, species strictly short-distance migrant; *NSBT*, number of birds of a particular species infested with ticks; *NTSB*, number of all tick species collected from a particular bird species; *I*., *Ixodes*; *L*, larvae; *N*, nymphs; *A*, adult; *PTI*, prevalence of tick infestation per bird species; *MIT*, mean intensity of tick infestation per bird

Tick infestation was observed in 19.5% (*n* = 8) of the captured birds. A detailed overview of tick infestation rates and the distribution of tick species in individual bird species included in the study is provided in Table 1.

The highest MIT was observed in Great Tits (4 ticks per bird), followed by Dunnocks (2.5 ticks per bird) and Common Blackbirds (2 ticks per bird) (Table 1).

In total, 19 ticks were collected from the birds. *Ixodes ricinus* ticks represented 89.5% (*n* = 17) of the collected samples, while *I. ventalloi* comprised 10.5% (*n* = 2) of the sample pool. In terms of developmental stages, nymphs of *I. ricinus* were predominant, constituting 84.2% (*n* = 16), followed by larvae at 5.3% (*n* = 1), with the remaining being adult females of *I. ventalloi* at 10.5% (*n* = 2) (Table 1).

*I. ventalloi* females (A – 438 and B – 439) were collected from two Dunnocks captured on 21 March 2021.

### Migratory route of infested Dunnocks (*P. modularis*)

**Fig. 1:**
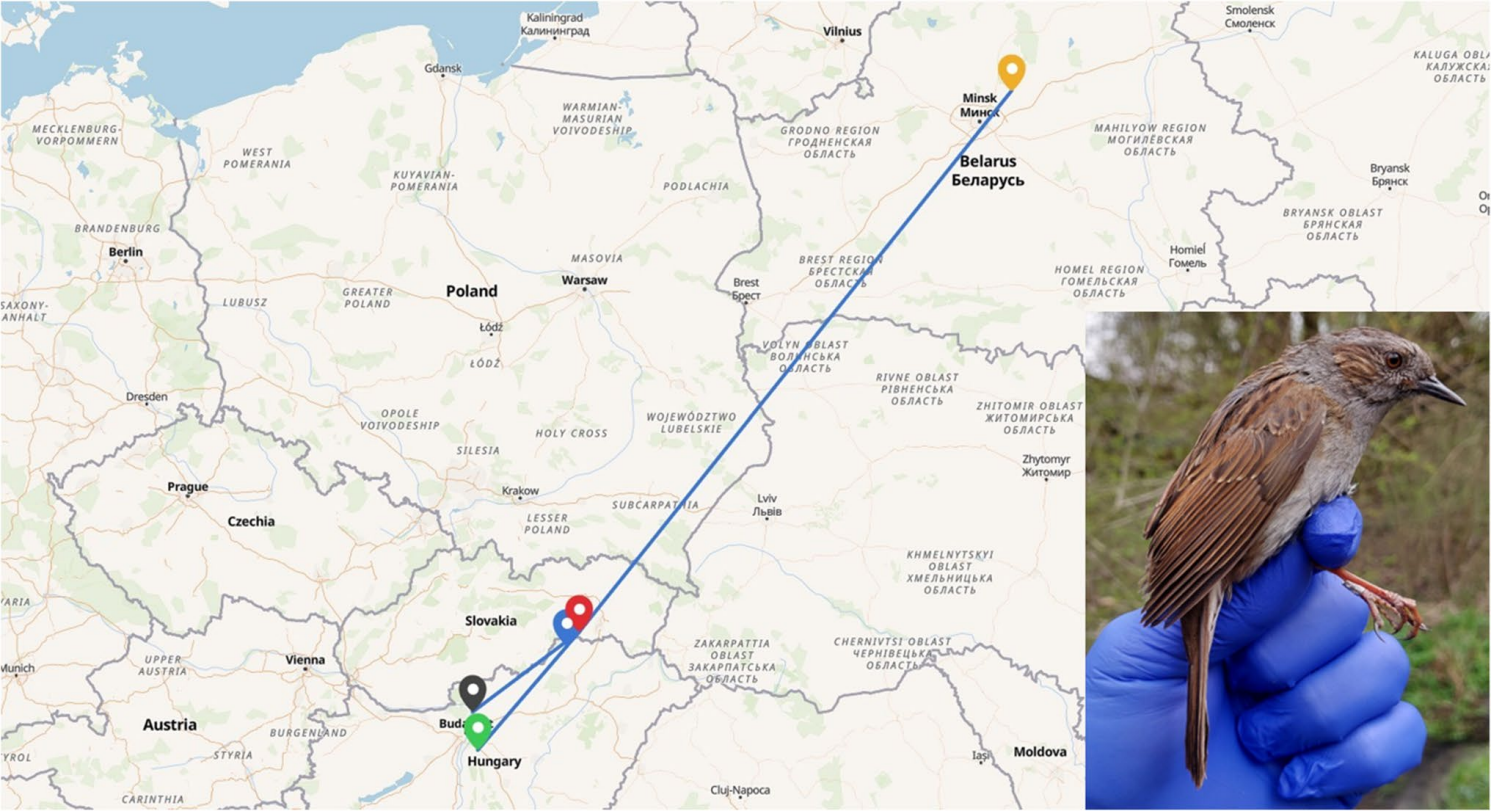
Movements of Dunnocks between Drienovec Bird Ringing Station, Slovakia (red point), Sutoki, Ringing Station Gorodishche, Belarus (orange point), Szalonna Bird Ringing Station, Hungary (blue point), Sződliget, Hungary (black point) and Ócsa, Hungary (green point). In the right corner of the map is a picture of a trapped Dunnock

The Dunnocks migrate through the Drienovská Wetland between the north-east and south-west Europe (Fig. 1). *P. modularis* does not belong to the breeding avifauna of the Drienovská Wetland. The results from bird ringing indicate that the Dunnocks transmigrating through the locality spend the winter season in the eastern Mediterranean or Italy. Dunnocks ringed at Bird Ringing Station Drienovec were later found in autumn and spring at sites in Hungary (Szalonna Bird Ringing Station, Ócsa and Sződliget). An exception is a bird ringed in October 2022 at Drienovec and later trapped in April 2023 at the ornithological station in Sutoki, Belarus.

### Ixodes ventalloi

#### Morphological identification

**Fig. 2:**
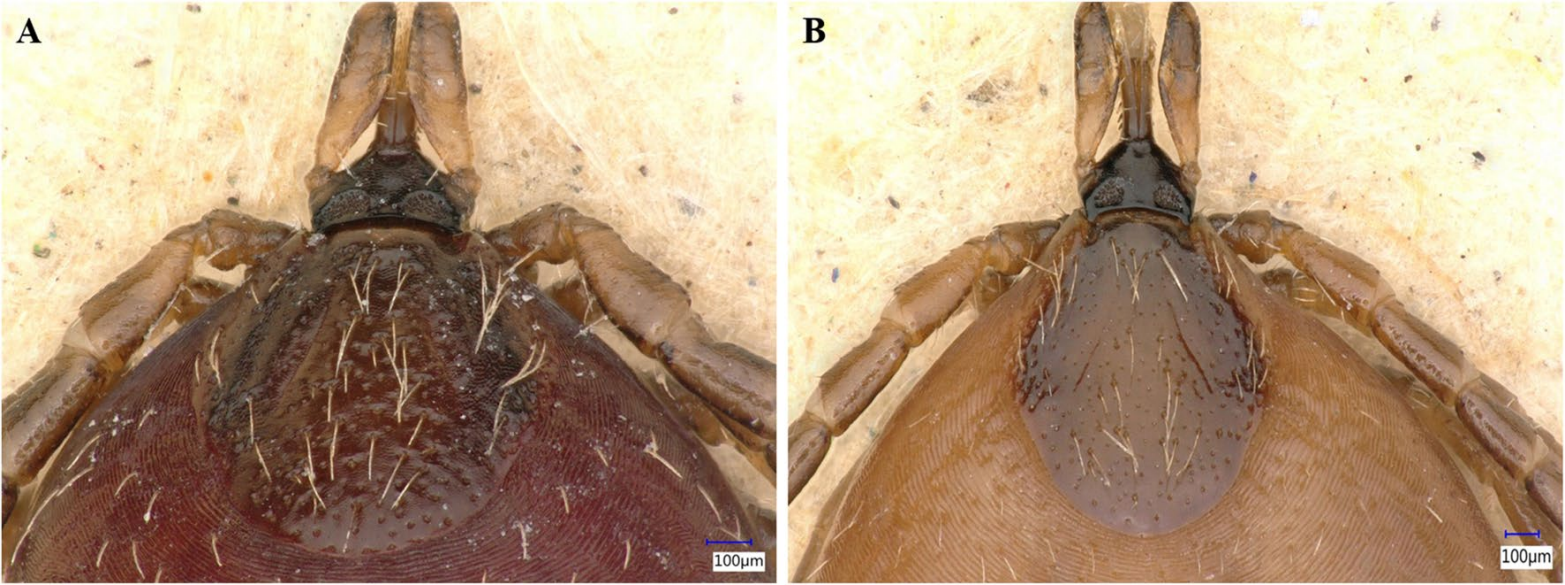
*Ixodes ventalloi* females dorsal view with detail on scutum and gnathosoma

**Fig. 3:**
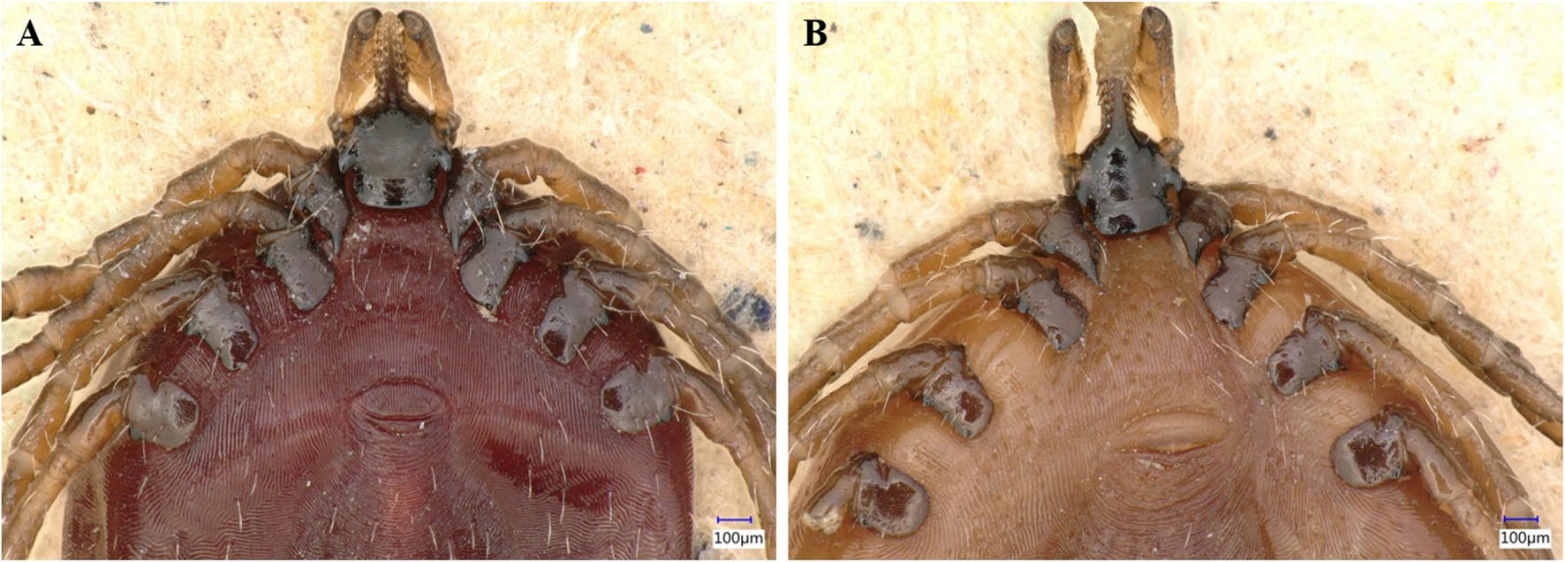
*Ixodes ventalloi* females ventral view with detail on coxae I–IV, genital aperture, and gnathosoma

**Fig. 4:**
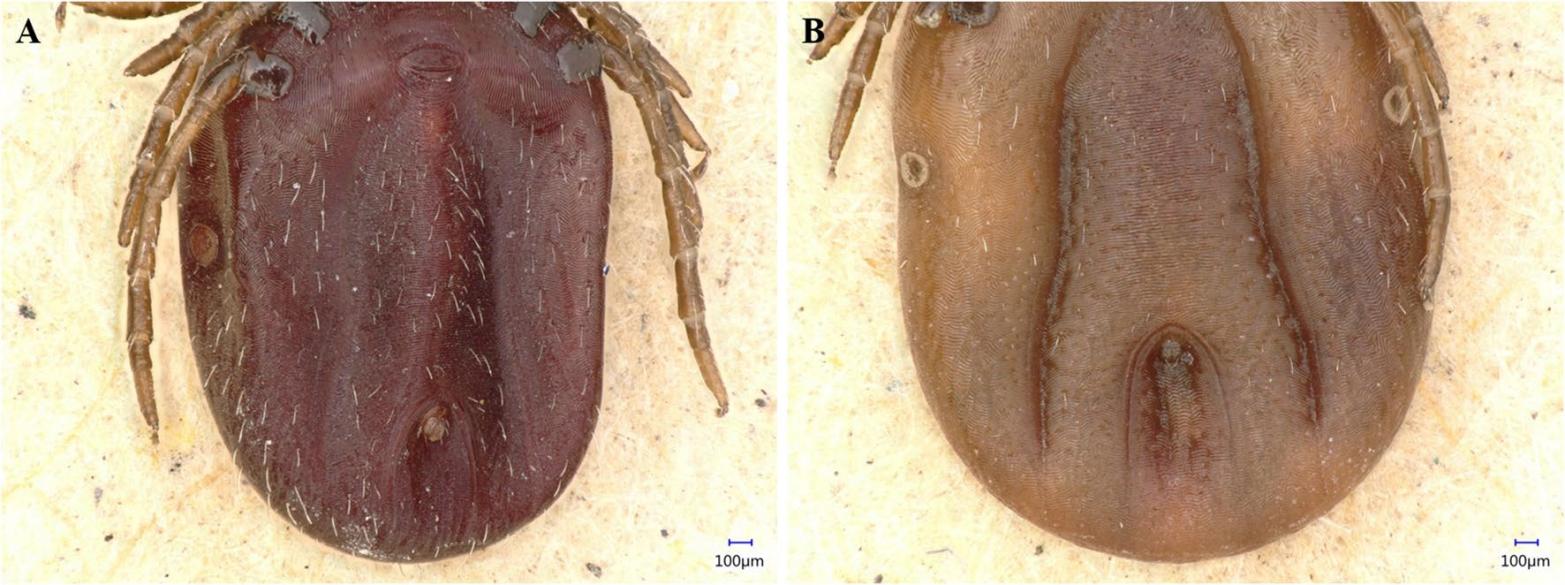
*Ixodes ventalloi* females ventral view with detail on spiracular plates, anal and genital grooves

*Ixodes ventalloi* females A (438) and B (439): Idiosoma: Female A has a scutum with a broadly rounded outline and distinct cervical grooves, with scattered punctations and numerous long setae, especially in the central area (Fig. 2A). The genital aperture is located between coxae III and IV (Fig. 3A), a notable dislocation, attributed to engorgement. The spiracular plate is oval (Fig. 4A). Legs are moderately long and slender (Fig. 4A). Coxae I–IV (Fig. 3A), each with a small external spur, with those on coxae II and III being the longest. Coxa I with long, straight, and pointed internal spur (Fig. 3A). The scutum of female B is very similar to that of female A, but with an ovoid outline (Fig. 2B). The genital aperture is also dislocated between coxae III and IV due to blood consumption (Fig. 3B). Spiracular plates, coxae, and legs resemble those of female A (Figs. 3B and 4B). However, female B has a shorter internal spur on coxa I than female A (Fig. 3B).

*Ixodes ventalloi* females A (438) and B (439): Gnathosoma: Female A has a posterior margin of the basis capituli dorsally broadly concave with small cornua (Fig. 2A). Porose areas are irregularly ovoid, anterior margin broadly rounded, the posterior margin essentially straight and well delineated; separated by a distance that is about half of each area (Fig. 2A). The basis capituli ventrally has a slightly rounded posterior margin, and the auriculae are very large, pointed, and less curved (Fig. 3A). Palps are long, with the apical part being rounded. Hypostome is long, featured by distinct hypostomal dentition with three rows of 2/2 on the basis and eight rows of 3/3 on the apex (Fig. 3A). Female B’s posterior margin of the basis capituli and palps are similar to female A, but the porose areas are triangular shaped and separated by a distance that is slightly larger than the breadth of each area (Fig. 2B). Pores in porose areas are irregularly placed in both females (Fig. 2). The basis capituli of female B has a straight posterior margin ventrally, the auriculae are very large, hook-like, and internally curved. Hypostome is not visible (Fig. 3B).

### Sequence identity and phylogenetic analyses

**Fig. 5:**
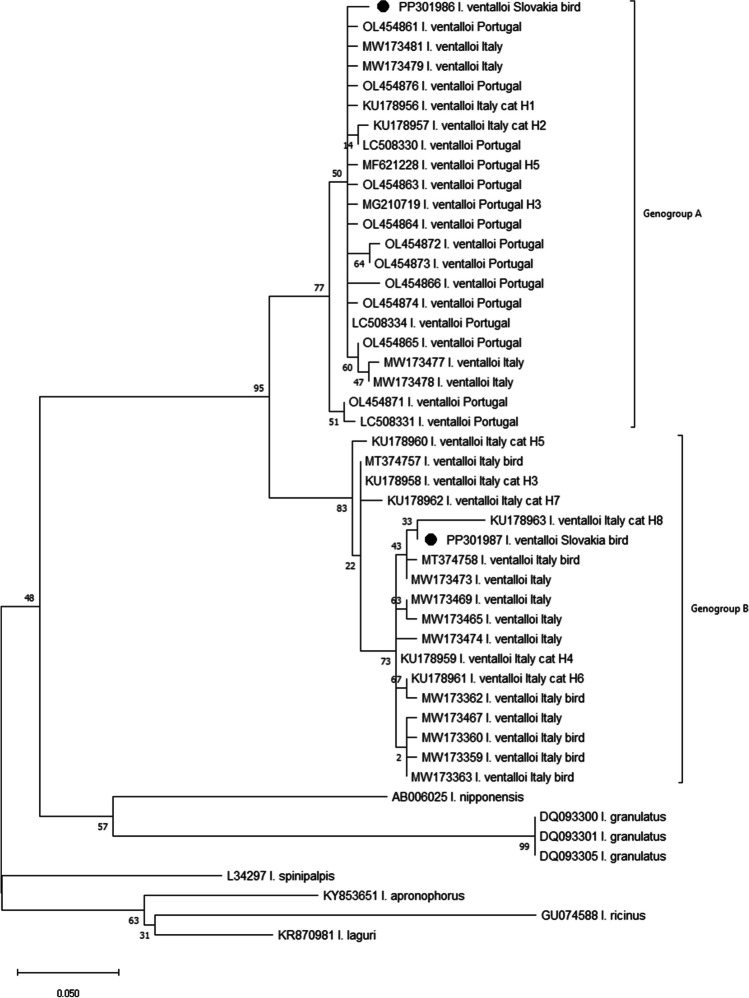
Phylogenetic tree based on mitochondrial 16S rRNA sequences obtained from *Ixodes ventalloi* collected in Italy, Portugal, and Slovakia, compared to sequences of other related *Ixodes* species available in GenBank. Strains from Slovakia are marked with a black dots (PP301986, PP301987). Accession numbers are followed by tick species name and in the case of *I. ventalloi* sequences, if possible, the origin of sequences and haplotype designation. Clusters are labelled according to Latrofa et al. (2017) and Santos and Santos-Silva (2018)

Phylogenetic analysis of the mitochondrial 16S rRNA gene sequences revealed that the newly identified Slovak specimens, designated as 438_16S and 439_16S, are clustered into distinct genogroups as defined by Latrofa et al. (2017) (Fig. 5). Specifically, within Genogroup A, the analysis demonstrates that isolate 438_16S (PP301986) created a separate branch similar to other isolates obtained mostly from vegetation in Portugal and Italy (Fig. 5). Conversely, isolate 439_16S (PP301987) cluster within Genogroup B, where it formed sister taxa with Italian *I. ventalloi* haplotype 8 obtained from a cat (KU178963). These taxa create a clade with *I. ventalloi* isolates MW173473 (IV-USZ260T) and MT374758 (A3), also from Italy. Interestingly, isolate A3, like 439_16S, was obtained from a tick (nymph) parasitizing short-distance migrant Black Redstart (*Phoenicurus ochruros*) (Fig. 5).

The average pairwise distance (Nucleotide: MCL) within Genogroup A was 0.01, and within Genogroup B, it was 0.02. The inter-genogroup average pairwise distance stood at 0.08.

Regarding nucleotide sequence identity, isolate 438_16S exhibited an identity range of 97 to 98.5% with other representatives of Genogroup A. The sequence identity of isolate 439_16S with members of Genogroup B ranged between 96.1 and 99.5%. The comparative analysis between isolates 438_16S and 439_16S revealed a nucleotide identity of 90.2% within the examined nucleotide fragment, highlighting the distinct genetic backgrounds of these isolates.

## Discussion

In this manuscript, we describe the first detection of *Ixodes ventalloi* in Slovakia. Moreover, this is the first detection of this tick species on Dunnocks (*Prunella modularis*).

Dunnocks are common songbirds endemic to the Western Palearctic. Migration patterns among these birds exhibit variance, with some populations from northern Europe undertaking brief migratory journeys to spend the winter in southern Europe. In contrast, the breeding populations established in these southern locales exhibit sedentariness, opting not to migrate (del Hoyo and Collar 2016). We hypothesize that these migratory behaviors are instrumental in the dispersal of *I. ventalloi* to regions such as Slovakia. Dunnocks are predominantly ground feeders and often shed leaves and pieces of soil in search of food (Bishton 1986). This foraging strategy is believed to contribute significantly to their susceptibility to tick infestations, which leads to their role as hosts of many tick-borne pathogens (Sparagano et al. 2015).

Genetic analysis shows that Slovak isolates (438_16S, 439_16S) belong to different *I. ventalloi* genogroups. Both *I. ventalloi* females were collected from Dunnocks in March, and results suggest that females from differing genogroups exhibit no variance in seasonal activity or host preference. Previous studies have noted the coexistence of these genogroups within identical geographical areas, but the reason for the different evolution of these genogroups remains unknown (Latrofa et al. 2017). It is hypothesized that the divergent genotypes originated in separate regions, and their concurrent presence in a single locality is coincidental, perhaps due to bird migration, paralleling the distribution patterns observed in *I. scapularis* (Krakowetz et al. 2011; Latrofa et al. 2017). Although traditionally classified as a “rabbit tick” (Estrada-Peña et al. 2017; Gilot and Perez 1978; González et al. 2016; Jameson and Medlock 2011), an increasing number of studies are reporting the occurrence of all developmental stages of *I. ventalloi* on avian hosts (Rollins et al. 2021; Santos-Silva et al. 2006; Toma et al. 2021). In total, *I. ventalloi* ticks have been recorded from 13 bird species belonging to four orders—Strigiformes: *Asio flammeus*, *A. otus*, *Athene noctua*, *Tyto alba* (Gil Collado 1936; Gilot and Perez 1978; Martin 1988; Santos-Silva et al. 2006, 2011); Passeriformes: *Pica pica*, *Turdus merula*, *T. pilaris*, *Erithacus rubecula*, *Phoenicurus ochruros* (Gilot and Perez 1978; Jameson and Medlock 2011; Norte et al. 2015; Rollins et al. 2021); Galliformes: *Alectoris chukar*, *Al. rufa*, *Phasianus colchicus* (Gilot and Perez 1978; Psaroulaki et al. 2014; Tomassone et al. 2013), Gruiformes: *Rallus aquaticus* (Tomassone et al. 2013).

While genetic analysis has validated the species identity, discernible morphological discrepancies among the *I. ventalloi* females were observed, indicating the potential existence of distinct morphotypes. This observation appears to challenge the statement of Latrofa et al. (2017), who suggested that each *I. ventalloi* specimen displays the same phenotype. Both females differ from the general description of *I. ventalloi* in several key features. Auriculae of female A (438) are less curved than those observed in female B (439); in addition, both females also differ in the shape of coxa I. Female A has coxa I with longer, straight, and pointed internal spur. In contrast, female B has coxa I with a relatively short internal spur. Differences were also observed in the shape and size of the porose areas. However, it should be noted that some of these differences may be due to manipulation, stage of engorgement, the way the females were collected and stored, or could represent morphological anomalies. To verify the existence of different morphotypes, further research with non-engorged specimens would be necessary. Nevertheless, these observations suggest that relying on morphological features alone to identify *I. ventalloi* may be insufficient and should be supported by mitochondrial DNA analysis using molecular markers such as 12S rRNA, 16S rRNA, or cytochrome c oxidase subunit 1 (cox1) (Estrada-Peña et al. 2018; Latrofa et al. 2017; Santos et al. 2018).

To date, in Slovakia, 24 tick species have been described, including *I. ventalloi* identified in our study (Bona and Stanko 2013; Capek et al. 2014; Černý 1972; Didyk et al. 2021; Karbowiak et al. 2020; Nosek et al. 1982; Rosický 1953; Stanko and Csanády 2022). From both veterinary and medical perspectives, the most important species are *I. ricinus*, *Dermacentor reticulatus*, *D. marginatus*, *Haemaphysalis inermis*, *H. concinna*, and *H. punctata*, which are responsible for the transmission of a wide range of pathogens (Stanko et al. 2022). The vectorial capacity of *I. ventalloi* remains to be fully clarified. However, the detection of pathogens (e.g., *Anaplasma phagocytophilum*, *Coxiella burnetii*, and Eyach virus) in various developmental stages and degrees of engorgement in *I. ventalloi* indicates a potential for infection through transstadial and horizontal transmission, as well as possibly through co-feeding (Antunes et al. 2016; Chastel et al. 1984; Ioannou et al. 2009; Latrofa et al. 2017; Márquez and Millán 2009; Pennisi et al. 2015; Santos et al. 2004, 2018; Santos-Silva et al. 2006). Furthermore, these ticks have demonstrated a much broader host range than previously believed, potentially enhancing their role as pathogens vector. In Portugal, *I. ventalloi* ticks were frequently found together with *I. ricinus* ticks, implying not only shared environmental conditions but perhaps also shared pathogenic profiles, thereby highlighting the significance of *I. ventalloi* in both veterinary and human medical contexts (Santos-Silva et al. 2011).

In Slovakia, the presence of *I. ventalloi* is likely accidental. This situation mirrors an event in Germany where the same tick species was found on birds and first described by Petney et al. (1996). However, since the initial discovery, there have been no further reports of *I. ventalloi* being found in Germany. Nevertheless, given the similar ecological requirements shared with *I. ricinus*—the most prevalent tick species in Slovakia—ongoing surveillance of *I. ventalloi* is imperative. Further research is needed to verify the establishment of sustainable populations of *I. ventalloi* in Slovakia, employing methodologies such as flagging or dragging to enhance understanding of the distribution of this species.

## Conclusion

We report here the first detection of *Ixodes ventalloi* in Slovakia and its first collection on Dunnocks (*P. modularis*), expanding the known host range and geographical distribution of this tick species. Genetic analyses reveal that Slovak *I. ventalloi* specimens belong to different genogroups, suggesting complex evolutionary dynamics similar to patterns seen in other tick species. Traditionally considered a “rabbit tick,” the increasing evidence of *I. ventalloi*’s association with avian hosts underscores its ecological adaptability. Our results also highlight the role of birds in the dissemination of *I. ventalloi* populations from their endemic regions. Furthermore, this study indicates morphological diversity among *I. ventalloi* females, challenging previous assumptions of uniform phenotypes and suggesting the presence of distinct morphotypes. These findings support the integration of molecular techniques with traditional morphological identification methods to ensure accurate species identification. Further research is needed regarding the establishment of *I. ventalloi* populations in Slovakia.

## Data Availability

The data and materials generated during the current study are available from the corresponding author upon reasonable request.
